# The Age-Related Course of COVID-19 in Pediatric Patients—1405 Cases in a Single Center

**DOI:** 10.3390/jcm11247347

**Published:** 2022-12-10

**Authors:** Lidia Stopyra, Aleksandra Kowalik, Justyna Stala, Ida Majchrzak, Justyna Szebla, Mateusz Jakosz, Przemko Kwinta

**Affiliations:** 1 Department of Infectious Diseases and Pediatrics, Zeromski Specialist Hospital in Krakow, 30-931 Kraków, Poland; 2 Department of Pediatrics, Andrzej Frycz Modrzewski Krakow University, 30-705 Kraków, Poland; 3 Department of Pediatrics, Jagiellonian University Medical College, 30-663 Kraków, Poland

**Keywords:** SARS-CoV-2, COVID-19, children, age, clinical presentation

## Abstract

Since the beginning of the pandemic, many reports have pointed to age as the most important risk factor for severe COVID-19 in adults, but this relationship is less clear in children. Between March 2020 and April 2022, 1405 pediatric COVID-19 patients were included in our prospective study, which aimed to analyze the disease’s characteristics in three age groups: infants, toddlers (1–5 years), and children (5–18 years). We observed male prevalence of the disease in infants and toddlers compared to female prevalence in children. Comorbidities appeared most often in children. In the first pandemic wave, the vast majority of pediatric patients were children, but later, the percentage of infant and toddler patients increased significantly. A total of 74% of hospitalized children were younger than five years. Upper respiratory tract symptoms were most common in infants and toddlers, and lower respiratory tract symptoms and gastroenterocolitis were more common in children. Neurological symptoms appeared similarly in all age groups. The activities of ALT, CK, and LDH were the most elevated in infants, along with D-dimers. The median length of hospitalization fluctuated between three and four days and was highest in infants. Severe courses were more common in adolescents.

## 1. Introduction

In December 2019, a cluster of severe, life-threatening cases of pneumonia was detected in the region of Wuhan in central China, and on 7 January 2020, the causative pathogen was identified as a novel RNA betacoronavirus. Because of its phylogenetic similarity to severe acute respiratory syndrome coronavirus (SARS-CoV), the new virus was named SARS-CoV-2 [1]. On 11 March 2020, the World Health Organization (WHO) announced the Coronavirus Disease 2019 (COVID-19) pandemic.

Many reports point to age as the most important risk factor for a severe course of COVID-19, and deaths in adults, while children were reported all over the world to present with an asymptomatic or mild course of the disease [2,3,4,5,6,7]. SARS-CoV-2-related death in children is rare. In the United States, as of 1 June 2022, there were >13 million cases of COVID-19 and 1533 COVID-19-associated deaths in children <18 years of age (<0.01%) reported to the CDC [8]. A small number of publications considered the problem of dependence between the course of COVID-19, the clinical presentation, the necessity for hospitalization, and age in pediatric patients. Their authors reported the prevalence in both young children and older children among hospitalized patients, meaning those with a more severe course of the disease [9,10,11,12,13,14]. Further investigation is necessary to understand the clinical presentation, laboratory findings, and indications for hospitalization, especially because of the changing COVID-19 course in pandemic waves caused by different SARS-CoV-2 variants [12,14,15,16,17].

This work aimed to analyze the COVID-19 characteristics of children in a single center, based on findings from 1405 patients aged 0 to 18 years that were hospitalized from 23 March 2020 to 30 April 2022. The analysis was conducted especially in terms of clinical presentation, laboratory findings, trends in pandemic waves, and outcomes in relation to age. Our observations may be useful for ongoing guidance for the evaluation, management, and prevention of COVID-19 for pediatric patients, depending on age.

## 2. Materials and Methods

On 23 March 2020, when the first children were admitted to the Department of Infectious Diseases and Pediatrics, this prospective study was started. The inclusion criteria were as follows: age 0–18 years, laboratory-confirmed COVID-19, and hospitalization. A total of 1405 children were included in this study. All of the children were symptomatic, and reinfections were included.

According to the World Health Organization (WHO) and the National Institute of Public Health recommendations [18,19], COVID-19 was diagnosed using a positive reverse transcription and real-time polymerase chain reaction (RT-PCR) and, since 30 October 2020, using the second-generation antigen tests based on a nasopharyngeal swab performed in certified laboratories. Several kits were used during the two years of the pandemic: (1) GeneFinder™ COVID-19 Plus RealAmp, Elitech, Biomedica, (2) Liferiver, Novel Coronavirus (2019-nCoV) Real Time Multiplex, (3) VIASURE CerTest, Biotec, (4) Maccura SARS-CoV-2 Fluorescent PCR, Maccura Biotechnology, (5) Homemade DIAGtest SARS-CoV-2 real time RT-PCR, and (6) Labsystems Diagnostics. In addition, we used the COVID-19 Real Time Multiplex RT-PCR and the second-generation Abbott Panbio-COVID-19 Ag Rapid Test Device (WHO laboratory 2020, AOTM).

The criteria for admission to the hospital were as per those for other pediatric diseases, e.g., dehydration, dyspnea, or seizures. According to the Polish Ministry of Health recommendations, hospitalization was compulsory for every patient with diagnosed SARS-CoV-2 infection up to September 2020 [20]. According to Polish expert group recommendations, hospital referrals were also required for children with congenital heart defects, neurologic diseases, genetic disorders, chronic renal diseases, mucoviscidosis, broncho-pulmonary dysplasia, immunodeficiency after organ transplantation, and diabetes mellitus. Newborns, infants, and children with obesity, especially those with a body mass index (BMI) > 30 kg/m^2^, were also included [21].

Discharge criteria were two negative PCR tests taken within 24 h. After 2 September 2020, the only criterion was the condition of the patient.

Data were collected and reported by the physicians working in the department according to a standardized questionnaire for the case history and physical examination that were obligatory for every patient. The standard laboratory tests for COVID-19 patients were taken for every hospitalized child with diagnosed COVID-19.

The questionnaire included the following:

Demographic and epidemiological data: (age, sex, ethnicity, nasopharyngeal/oral swabs for SARS-CoV-2 PCR obtainment date, recent contact with known COVID-19 patients, background illnesses including heart disease, chronic lung disease, and/or asthma, developmental delay, diabetes, immune compromise, and malignancy).

Signs and symptoms: fever, cough, rhinitis, dyspnea, sore throat, weakness, diarrhea, abdominal pain, vomiting, headache, conjunctivitis, nausea, myalgia, rash, ageusia, anosmia, chest pain, irritability, seizures, and headache. Anosmia/ageusia are difficult to ascertain for infants and toddlers. In the study it was assumed that, if an infant or toddler with COVID-19 completely refused to eat for several consecutive days, and other reasons such as severe condition and stomatitis were excluded, the patient had anosmia/ageusia.

Disease outcome data: the length of hospitalization, the necessity of oxygen treatment, the necessity of intravenous dehydration, antiviral treatment, systemic steroid therapy, and PICU admission.

Laboratory data: complete blood count (CBC) parameters, C-reactive protein (CRP), alanine transaminase (ALT), lactate dehydrogenase (LDH), creatinine kinase (CK), ferritin, vitamin D3 level, prothrombin time, D-dimers, nasal swabs for other viral pathogens (co-infection), and imaging (i.e., lung ultrasound (LU), chest X-ray, and high-resolution computed tomography (HRCT)).

Final diagnoses: upper respiratory tract infection, lower respiratory tract infection, gastroenterocolitis, neurological diagnoses, and others.

Radiographic and USG pneumonia diagnoses were based on the interpretation by the treating physician. Lower respiratory infections were diagnosed based on clinical presentation and lung ultrasonography (LU), chest X-rays, and high-resolution computed tomography (HRCT). The most often administered examination, especially in the youngest children, was LU. The presence of focal, multifocal, and confluent B lines and pleural irregularities were the most common LU findings used to diagnose COVID-19 pneumonia. In chest X-ray examinations, bilateral and multifocal lesions were found most frequently, especially in the lower lobes. A pure ground-glass appearance was also typical of COVID-19 lower respiratory-related findings. Gastrointestinal infection was diagnosed based on clinical presentation (vomiting, diarrhea) and the exclusion of other etiologies such as rotaviruses, adenoviruses, and noroviruses.

To assess the COVID-19 characteristics depending on age, all 1405 hospitalized pediatric patients were assigned to three study groups: 567 infants (0–12 months), 470 toddlers (1–5 years), and 368 children (5–18 years).

The statistical analysis was performed using SPSS ver. 27 software (Armonk, NY, USA). Results were presented based on the parameters of descriptive statistics, including the mean values and standard deviations (SD) for the quantitative variables with a normal distribution or median values with the interquartile range for non-normally distributed data. Categorical variables were presented as numbers with percentages. Qualitative values were compared by the chi-square test. The Kruskal–Wallis test was used for the analysis of the continuous variables investigated in the study. In all cases of statistical significance, a pairwise comparison between the groups was performed using a post-hoc test. In all analyses, a *p*-value < 0.05 was considered statistically significant.

The study was performed in accordance with the ethical standards of the Declaration of Helsinki and its later amendments. It was approved by the Ethics Committee of the Regional Medical Chamber in Krakow No OIL/KBL/18/2020 on 10 March 2020.

## 3. Results

**Figure 1:**
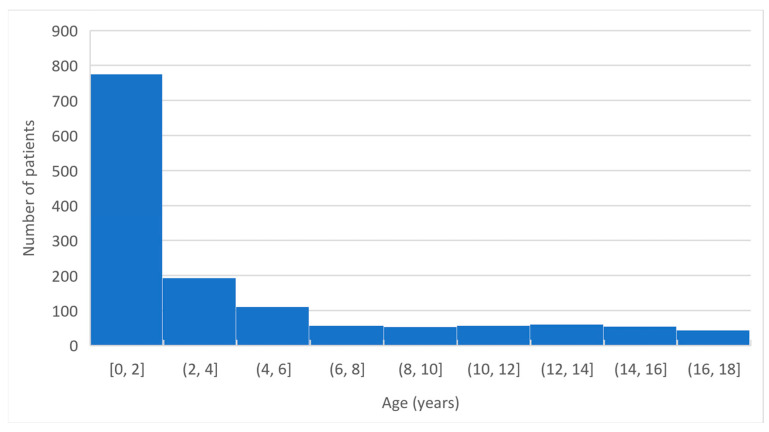
The age structure of the hospitalized children.

The age of the hospitalized children is presented in Figure 1. The high prevalence of infants younger than six months of age (26%) is noteworthy.

### 3.1. Study Groups

The demographic characteristic data are presented in Table 1.

The male sex was dominant in the groups of infants and toddlers, with the opposite finding for older children. However, the differences were not statistically significant. Only one child was South Asian; the rest (*n* = 1404) were Caucasian.

Immunocompromised patients, as well as children with chronic diseases at greater risk of severe COVID-19 (neurological disorders and pulmonological, cardiovascular, genetic, and oncologic diseases), were more common in the oldest study group than in the toddler and infant groups. A lack of BCG vaccination was more frequent in infants than in toddlers and children.

The course of COVID-19 in all the study groups was analyzed in each of the first five pandemic waves caused by different SARS-CoV-2 variants. In Poland, the first wave of the COVID-19 pandemic occurred from March to June 2020, the second from October 2020 to January 2021, and then variants were not reported in Poland, and SARS-CoV-2 sequencing was performed only occasionally. In the third wave from February to May 2021, the alpha (B.1.1.7) variant dominated, in the fourth wave from 1 October 2021 to 15 January 2022, the delta (B.1.617.2) variant dominated, and in the fifth wave from 16 January to 30 April 2022, the omicron variant (B.1.1.529, BA.1, BA.1.1, BA.2, BA.3, BA.4, and BA.5 lineages) dominated.

**Figure 2:**
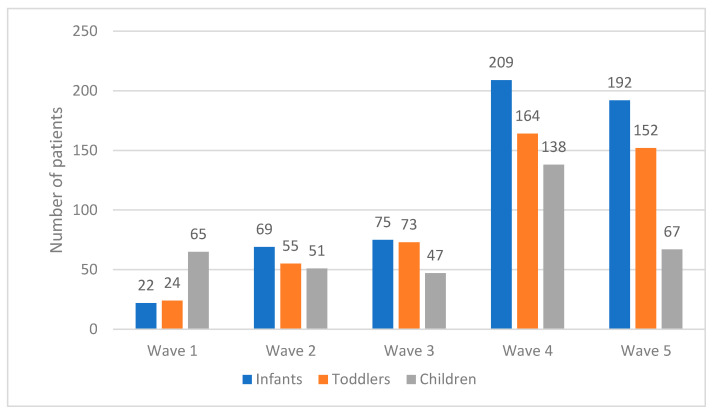
The number of patients in the groups of infants, toddlers, and children in the first five waves of the pandemic.

Regarding the age groups affected during the particular COVID-19 pandemic waves, the greatest differences were found between the first and fifth waves (Figure 2). In the first wave, the majority of patients were older than five years (65/111 (58.5%) versus 22/111 (19.8%) in the infant group and 24/111 (21.6%) in the toddler group (*p*-value < 0.001)). In the fifth wave, the group of patients consisted of 192/411 (46.7%) infants, 152/411 (37%) toddlers, and 67/411 (16.3%) children older than five years. In the other waves, the differences between age groups were statistically insignificant, but it is noteworthy that, in the subsequent pandemic waves, the median age of the hospitalized patients decreased significantly.

### 3.2. Clinical Presentation

The clinical presentation of COVID-19 in the study groups is presented in Table 2. In all age groups, fever was the most common sign of COVID-19, and it was most frequent in toddlers. As for respiratory symptoms, rhinitis was most common in infants and was rarely observed in children older than five years. Cough was less frequent in children than in the other two groups. Dyspnea was present in 11–15% of hospitalized children, with no significant differences between age groups. Anosmia and ageusia, the most typical symptoms of COVID-19, were rarely observed, and the differences between study groups were statistically insignificant. Neurologic symptoms (for example, seizures, paresis, vertigo) were most common in the children group.

### 3.3. Laboratory Findings

A comparison of laboratory findings in all the study groups is shown in Table 3 and Table S1. C-Reactive Protein (CRP) was significantly higher in the group of toddlers. Leukopenia and thrombocytopenia were most frequently observed in older children. Significant differences between the groups were noted in the levels of parameters such as alanine transaminase, creatinine kinase, lactate dehydrogenase, and D-dimers. All of these parameters were highest in the infant group.

### 3.4. COVID-19 Outcome

The COVID-19 outcome in the three study groups according to age was estimated based on four parameters: the necessity of oxygen therapy, intravenous rehydration treatment, general steroid therapy, and the length of hospitalization. The data are presented in Table 4.

Considering the fact that the study referred to hospitalized patients, the number of children requiring oxygen therapy was very low (2–5%) and was significantly the highest in the group of children older than five years. Intravenous rehydration was most frequently necessary in the group of toddlers, as was steroid therapy. The median length of hospitalization fluctuated between three and four days and was highest in infants.

A total of 14/1405 (1%) of our patients were treated with remdesivir based on Food and Drug Administration (FDA) and European Medicine Agency (EMA) recommendations [22,23]. Four of these patients were infants (1, 2, 8, and 11 months of age), and ten were children older than five years (9, 9, 12, 12, 15, 15, 15, 16, 16, and 18 years). A total of 3/1405 (0.2%) were treated with baricitinib according to FDA recommendations [24]. They were 7, 12, and 16 years of age.

### 3.5. Final Diagnoses

The most common diagnoses at the time of discharge from the hospital were upper and lower respiratory tract infections, gastroenterocolitis, and neurological syndromes. The differences between the age groups are presented in Table 5. The diagnosis of upper respiratory tract infection was most frequently made in the group of infants, and most patients suffered from laryngitis. We also observed otitis media in the course of COVID-19.

Lower respiratory tract infection was rarer in the group of infants in comparison with the other groups. Pneumonia in teenagers resembled that in adults. The prevalence of gastroenterocolitis was highest in the group of children.

Neurological diagnoses were made in 9–13% of the pediatric patients, with no statistically significant differences between the study groups.

The most common other diagnoses were urinary tract infections, hepatitis, and skin lesions.

## 4. Discussion

From the beginning of the pandemic, age was reported as one of the most important risk factors for a severe course, hospitalization, and death due to COVID-19, which was confirmed in adults. However, regarding pediatric patients, some authors reported that the youngest children were most threatened by COVID-19 [15,16,25], and others reported that teenagers were more at risk [26,27,28].

### 4.1. Demographic Characteristics

Although children, if compared to adults, have a lower risk of a severe course of COVID-19, 1405 children were hospitalized and met the inclusion criteria for this analysis. It is noteworthy that the youngest children most commonly required hospitalization (Figure 1). In our study, patients in the first year of life constituted 40.35% of the sample, and children younger than five years accounted for 73.8% of the hospitalized patients with COVID-19 in this study. To the best of our knowledge, this is one of the first large single-center studies concerning only hospitalized pediatric patients with COVID-19 over two years of the pandemic. Other authors reported both hospitalized and ambulatory-treated children infected with SARS-CoV-2 and did not observe a predominance of the youngest children [15,17,29,30]. The results from the studies concerning inpatients and outpatients with COVID-19 were similar: 2.4–3.8% of children in the 0–1-year group and 39.1–35.9% in the 0–5-year group, according to the pandemic wave, were reported by Murugan et al. [29]. A total of 6.1% of patients in the 0–1 year group and 25.2% in the 0–5-year group were reported in Krajcar’s study [17], and 35.1% of children aged 0–5 years were reported from Brazil [30]. A total of 45% of children in the 0–5-year group was reported in the SARSTer-PED study [15]. Among the hospitalized children, a huge prevalence of the youngest has been observed. Alteri reported from the period of the first four waves of the pandemic that, similar to our study results, 35.1% of hospitalized children with COVID-19 were 0–1 years old and 70.6% were 0–5 years old [16]. Swan reported from the United Kingdom (UK) that 52% of hospitalized children were 0–5 years old [31]. Recent CDC reports indicated that 22.5% of children hospitalized with COVID-19 were in the age range of 0–1 years, and 40.8% were aged 0–5 years [32]. There was also a significant increase in the percentage of pediatric patients younger than five years in hospital departments after the vaccination of children older than five years started [33]. Göktug observed in the Pediatric Emergency Department that 14.8% of patients were in the age range of 0–1 years compared to 32.4% in the 0–5-year range [27]. In our department, the predominance of the youngest children was much greater than that in the other reports, and we observed that, during the pandemic, the predominance of the youngest groups of children among hospitalized patients increased (Figure 2). Similar findings were reported by other authors [5,6,8]. Therefore, a difference between our study and those conducted previously was that this is the first study concerning a long period of time over the pandemic, from March 2020 to April 2022. This is also the reason for Alteri making this same observation. The increasing number of infants and toddlers among hospitalized patients with COVID-19 was probably due to the lifting of sanitary restrictions and the commencement of vaccination against COVID-19 for children older than five years, which caused a milder course and rarer need for hospitalization in older age groups. It is also noteworthy that patients with comorbidities and with immunodeficiency were the most numerous in the group of children older than five years, while the infants and toddlers often required hospitalization even though they had no chronic underlying conditions. This may be because the recommendations pointed to newborns and infants as being in the risk group for a severe course of COVID-19, causing more referrals of the youngest children to the hospital [21].

Comorbidities associated with the increased risk of severe disease mainly affected children older than five years (42.3%). Other researchers confirmed the same [34,35,36]. CDC reports also showed more underlying conditions among hospitalized pediatric patients aged 5–17 years (53.4%) and also, contrary to our findings, of 42.3% of patients in the youngest group (0–2 years of age) [37].

The differences between age groups in consecutive pandemic waves were of particular interest. The first wave was the only one in which the child group was the most numerous. In the next waves, the percentage of children decreased significantly. This was probably caused by the current Polish Health Ministry regulations. During the first wave and at the beginning of the second wave, hospitalization was obligatory for every patient who tested positive for SARS-CoV-2 by PCR. Only symptomatic patients were tested, and because of the strict lockdown, infants and toddlers were tested very rarely. At the beginning of the pandemic, the youngest children were infected the most often from a family member, while exposure other than household contacts was confirmed more frequently in teenagers [15]. Positive parents were isolated; they were not allowed to leave their houses, and there was no ability to have infants and toddlers tested up to the time hospitalization was necessary because of severe symptoms. In the fourth and fifth waves, vaccination against COVID-19 was possible for children older than five years, and it also had an influence on the age of hospitalized children [19,20,21].

The Bacillus Calmette-Guerin (BCG) vaccination parameter was also analyzed in this study (Table 1). This was due to the fact that, during the COVID-19 pandemic, it was hypothesized that countries without widespread tuberculosis prevention policies would have a higher percentage of severe disease course (Italy, France, Spain) than countries with long-term widespread prevention (Japan, Denmark, Korea) [38]. In Poland, according to The Obligatory Vaccination Schedule, the BCG vaccination is performed in the first days of life, which means that it was available to all children included in the study. The highest number of unvaccinated BCG patients was the group of infants whose disease severity required admission to the hospital, although they had the fewest underlying medical conditions. Nevertheless, fewer infants may be vaccinated against BCG in the general population. This needs further investigation.

### 4.2. Clinical Presentation and Final Diagnoses

The clinical presentation of COVID-19 in hospitalized children was multisymptomatic in all age groups. The most frequent symptoms were fever, cough, and rhinitis, especially in infants and toddlers. These symptoms were also described by other authors [25,32,39,40,41,42,43]. In infants, cough and dyspnea were more common symptoms of upper respiratory tract infection, and the most common symptom was laryngitis (Table 5). We also observed otitis media in the course of COVID-19. Similar observations were reported by other researchers [25]. High fever, vomiting, and diarrhea were observed much more frequently in patients aged 0–5 years. These symptoms influenced the need for hospitalization and intravenous rehydration but did not cause a severe and life-threatening course of the disease. In many previous publications, the authors reported gastrointestinal symptoms (vomiting, diarrhea, and abdominal pain) as typical for pediatric patients and more often in younger children [15,25,28,31,39,40,42,44]. Nevertheless, there are some publications reporting a low percentage of gastrointestinal symptoms in hospitalized children [41,45]. Because these same signs and symptoms can appear in different diseases, we also analyzed the diagnoses at discharge from the hospital. This seems to be important because, e.g., vomiting can appear in the course of gastroenterocolitis but also in neurological diseases and even during pneumonia and upper respiratory tract infections. It is interesting that gastroenterocolitis in our cohort was diagnosed as most common in children older than five years. Previous research usually did not consider final diagnoses, focusing on signs and symptoms; thus, it is not possible to compare our observations to those in other studies.

Neurologic symptoms were significantly more common in the group of children older than five years, similar to other authors’ reports [46,47].

Based on statistical data, it seems that anosmia and ageusia are more common in children older than five years, but this is difficult to analyze because toddlers and infants cannot verbalize such symptoms. It is noteworthy that, in our study, infants and toddlers most often required intravenous rehydration, although we did not observe relevant ongoing fluid losses. This might have been because of anosmia and ageusia. Many authors confirmed that the main problem is that symptoms related to taste and smell are so subjective that it is difficult to assess them in pediatric age groups [48]. Nevertheless, Yan et al., in an analysis of 18 eligible studies, also concluded that higher smell or taste dysfunction rates were associated with a younger age [49].

### 4.3. Laboratory Findings

Differences between the three studied groups were found in the laboratory test results (Table S1 and Table 3). Most abnormalities appeared in the infant group; they were temporary and did not differ significantly from those of other viral diseases. Although changes in laboratory results have been widely described in adults and in children with COVID-19, there are only a few studies concerning laboratory findings in hospitalized pediatric patients according to age. Similar to our observations, they have all reported leucopenia, neutropenia, lymphocytosis, thrombocytopenia, and elevated CRP, CK, LDH, ALT, and D-dimers as typical in most pediatric patients with COVID-19, especially in patients 0–5 years old [29,40,41,50,51]. Nevertheless, some publications show a relationship between the laboratory parameters at admission and hospitalization and the patient’s prognosis at every age. They showed that severe cases had significantly higher levels of mean serum lactate, CRP, ALT, and D-dimers, and an increase in LDH was observed even in asymptomatic patients [40,45,51].

### 4.4. Disease Outcome

According to other reports, 9–54% of hospitalized pediatric patients with COVID-19 required oxygen support [29,32,44,52], while, in our department, only 2% of infants and toddlers and 5% of children older than five years required this intervention. This difference could have been because infants were hospitalized with a much milder course of COVID-19 than older children because of the recommendations that infants be considered at a high risk of a severe course of the disease [21]. One patient in our department required high-flow nasal oxygen therapy (HFNOT), only three (0.2% of our cohort, 0.8% of the children group) had to be referred to the Pediatric Intensive Care Unit (PICU), and no patients died. All four patients with the most severe course of COVID-19 were older than 10 years, and all had chronic diseases (cerebral palsy, genetic disorders, and critical obesity). These data significantly departed from those of other authors, who reported that up to 21% of patients required PICU admission [28,31,45]. This can be explained by the higher percentage of infants and toddlers in our cohort and by the fact that almost all of our patients were of white Caucasian ethnicity, while in numerous reports, it was revealed that greater disease severity was associated with Black or other non-White races and ages older than four years [31,53,54].

The median length of hospitalization in our cohort was 3–4 days and was significantly longer in the group of infants. In other publications, the authors reported shorter hospitalization. Swann from U.K. and Cloete from South Africa reported an average two-day stay [31,44]. Other authors reported much longer stays exceeding 10 days [11,40]. Hospitalization was significantly longer in the group of infants, similar to other authors’ reports [11,15,25,40].

Our study had several limitations. At the beginning of the pandemic, primary care for COVID-19 patients was limited; thus, they were often referred to the hospital. The recommendations regarding the rules for COVID-19 testing, isolation, and hospitalization changed, and this could have influenced the admission criteria and the length of hospitalization. Our experience with pediatric COVID-19 also increased during the pandemic, which could have influenced hospital admissions and the length of stay.

To the best of our knowledge, this is the first large single-center study comparing the differences between the clinical course of pediatric hospitalized COVID-19 patients in different age groups for more than two years of the pandemic.

## 5. Conclusions

Many statistically significant differences in disease signs and symptoms were observed between study groups. Infants were the most often hospitalized patients in the first two years of the pandemic. The reasons for infant hospitalization were the necessity of intravenous rehydration because of dehydration in the course of high fever, vomiting and diarrhea, and appetite disorders.

Severe courses requiring oxygen supplementation and antiviral therapy were more common in adolescents.

A total of 74% of the hospitalized patients with COVID-19 were younger than five years, with a growing trend in the subsequent waves, most likely due to vaccination rolling out to the older age groups.

## Figures and Tables

**Table 1:** Demographic characteristics of hospitalized COVID-19 pediatric patients in the infant, toddler, and children groups.

	Infants (*n* = 567)	Toddlers (*n* = 470)	Children (*n* = 368)	*p*-Value
Male sex: *n* (%)	313 (55.3)	254 (54.2)	173 (47.5)	0.054
Patients with chronic diseases: *n* (%)	85 (15.2)	119 (25.4)	153 (42.3)	<0.001
Immunocompromised patients: *n* (%)	4 (0.7)	4 (0.9)	13 (3.6)	0.001
BCG unvaccinated patients: *n* (%)	23 (4.2)	12 (2.6)	5 (1.4)	0.047

BCG: Bacillus Calmette-Guerin.

**Table 2:** Clinical characteristics of COVID-19 in infants, toddlers, and children.

	Infants (*n* = 567)	Toddlers (*n* = 470)	Children (*n* = 368)	*p*-Value
Fever: *n* (%)	389 (69)	361 (77)	230 (63)	<0.001
Rhinitis: *n* (%)	295 (52)	201 (43)	112 (31)	<0.001
Cough: *n* (%)	317 (56)	273 (58)	178 (48)	0.014
Dyspnea: *n* (%)	61 (11)	50 (11)	56 (15)	0.080
Vomiting: *n* (%)	84 (15)	134 (29)	69 (19)	<0.001
Diarrhea: *n* (%)	126 (22)	129 (27)	56 (15)	<0.001
Anosmia *n* (%)	29 (5)	18 (4)	28 (8)	0.057
Ageusia: *n* (%)	30 (5)	18 (4)	26 (7)	0.12
Neurologic symptoms: *n* (%)	22 (4)	36 (8)	75 (21)	<0.001

**Table 3:** Laboratory findings in hospitalized pediatric patients with COVID-19 according to age, based on the chi-square test.

	Infants (*n* = 567)	Toddlers (*n* = 470)	Children (*n* = 368)	*p*-Value
CRP > 5 mg/dL	129 (24)	224 (51)	154 (46)	<0.001
Leukocytes (10^3^/μL)				<0.001
<4.5	29 (5)	32 (7)	88 (26)	
4.5–13.5	427	328	229	
>13.5	79 (15)	84 (19)	24 (7)	
Neutrophils (10^3^/μL)				
<1.0	127 (25)	24 (6)	20 (6)	
1.0–6.5	359	308	259	
>6.5	30 (6)	99 (23)	50 (15)	
Lymphocytes (10^3^/μL)				
<1.0	8 (2)	29 (7)	59 (18)	
1.0–7.0	373	371	266	
>7.0	136 (26)	32 (7)	1 (0.3)	
Blood platelets < 100,000/μL	4 (0.7)	9 (2)	10 (3)	0.050
Alanine transaminase > 54 U/L	48 (9)	13 (3)	13 (4)	<0.001
Creatinine kinase < 170 U/L	123 (28)	49 (13)	23 (8)	<0.001
Lactate dehydrogenase > 220 IU/L	454 (97)	335 (87)	105 (35)	<0.001
D-dimers > 500 ng/mL	305 (76)	161 (44)	106 (36)	<0.001

CRP: C-reactive protein.

**Table 4:** The disease outcome for 1405 hospitalized pediatric patients with COVID-19 according to age.

	Infants (*n* = 567)	Toddlers (*n* = 470)	Children (*n* = 368)	*p*-Value	Post-Hoc Analysis
Oxygen therapy: *n* (%)	11 (2)	10 (2)	17 (5)	0.043	
Intravenous fluids: *n* (%)	202 (36)	233 (50)	154 (42)	<0.001	
Steroid therapy: *n* (%)	28 (5)	49 (10)	27 (7)	0.004	
Length of stay (days) Median (25th–75th percentile)	4 (2–5)	3 (2–4)	3 (2–5)	0.021	1 vs. 2:0.006

**Table 5:** Final diagnoses in hospitalized pediatric patients with COVID-19 according to age.

	Infants (*n* = 567)	Toddlers (*n* = 470)	Children (*n* = 368)	*p*-Value
Upper respiratory tract infection	355 (63)	280 (60)	188 (51)	0.002
Lower respiratory tract infection	137 (24)	140 (30)	124 (34)	0.005
Gastroenterocolitis	79 (14)	81 (17)	77 (21)	0.019
Neurological diagnoses	50 (9)	48 (10)	49 (13)	0.090
Other	101 (18)	82 (18)	69 (19)	0.881

## Data Availability

The datasets used and analyzed during the current study are available from the corresponding author upon reasonable request.

## Supplementary Materials

The following supporting information can be downloaded at: https://www.mdpi.com/article/10.3390/jcm11247347/s1, Table S1: Laboratory findings in hospitalized pediatric patients with COVID-19 according to age, based on the Kruskal–Wallis test.
