# Supplementary material for: The Age-Related Course of COVID-19 in Pediatric Patients—1405 Cases in a Single Center
Source: J Clin Med. 2022 Dec 10;11(24):7347. doi: 10.3390/jcm11247347 (PMC9782360; doi:10.3390/jcm11247347)
Supplement: Supplementary file 1 [file jcm-11-07347-s001.zip › jcm-2062863-supplementary.pdf]

Table S1. Laboratory findings in hospitalized pediatric patients with COVID-19 according to age, based on the Kruskal–Wallis test.

	**Infants (*n* = 567)**	**Toddlers (*n* = 470)**	**Children (*n* = 368)**	***p*-Value**	**Post-hoc analysis**
CRP [mg/dL]	1.7 (1–4.9)	5.2 (1.3–19)	3.65 (1–14)	<0.001	1 vs. 2: <0.001<br>1 vs. 3: <0.001<br>2 vs. 3: 0.005
Leukocytes [10^3/μL]	8.8 (6.5–11.7)	8.6 ([6.2–12.2)	6.2 (4.4–8.4)	<0.001	1 vs. 3: <0.001<br>2 vs. 3: <0.001
Neutrophils [10^3/μL]	1.81 (1–3.2)	3.63 (2.2–6.2)	3.2 (2.1–5.0)	<0.001	1 vs. 2: <0.001<br>1 vs. 3: <0.001<br>2 vs. 3: 0.018
Lymphocytes [10^3/μL]	5.3 (3.4–7.3)	3.4 (2.2–4.7)	1.8 (1.1–2.5)	<0.001	1 vs. 2: <0.001<br>1 vs. 3: <0.001<br>2 vs. 3: <0.001
Blood platelets [10^3/μL]	333 (273–412)	273 (220–346)	228 (180–288)	<0.001	1 vs. 2: <0.001<br>1 vs. 3: <0.001<br>2 vs. 3: <0.001
Alanine transaminase [U/L]	28 (21–38)	16 (12–22)	14 (11–20)	<0.001	1 vs. 2: <0.001<br>1 vs. 3: <0.001
Creatinine kinase [U/L]	125 (93–180)	100 (67–141)	73 (52–104)	<0.001	1 vs. 2: <0.001<br>1 vs. 3: <0.001<br>2 vs. 3: <0.001
Lactate dehydrogenase [IU/L]	311 (275–346)	280 (243–313)	200 (172–234)	<0.001	1 vs. 2: <0.001<br>1 vs. 3: <0.001<br>2 vs. 3: <0.001
D-dimers [ng/mL]	760 (517–1298)	466 (313–798)	359 (256–716)	<0.001	1 vs. 2: <0.001<br>1 vs. 3: <0.001<br>2 vs. 3: 0.004
